# Using loop-primer mediated PCR to enhance the detection of poorly preserved DNA

**DOI:** 10.3389/fgene.2022.1000123

**Published:** 2022-11-30

**Authors:** Hai Xiang, Zhi Wang, Liu Yang, Xing Zhang, Xingbo Zhao

**Affiliations:** ^1^ Guangdong Provincial Key Laboratory of Animal Molecular Design and Precise Breeding, School of Life Science and Engineering, Foshan University, Foshan, China; ^2^ National Engineering Laboratory for Animal Breeding, Key Laboratory of Animal Genetics, Breeding and Reproduction, Ministry of Agriculture and Rural Affairs, College of Animal Science and Technology, China Agricultural University, Beijing, China

**Keywords:** loop-primer, L-PCR, nested PCR, ancient DNA (aDNA), poorly preserved DNA

## Abstract

Ancient DNA is vitally important in evolutionary research, and obtaining authentic ancient DNA sequences is critical for a proper analysis. However, it is difficult to acquire amplicons accurately and efficiently from ancient DNA templates using current techniques. Here, we established a loop-primer-mediated amplification method (L-PCR) to obtain target ancient DNA sequences with high accuracy and efficiency. The method was tested using 66 ancient samples (including 27 pig bones or teeth and 39 chicken bones) and serially diluted modern animal DNA templates. Compared to nested PCR, L-PCR was proven to be more efficient and accurate and could obtain more amplicons from both ancient pig samples and chicken bones and detect as low as 10^−3^ ng/μl modern pig template DNA. The efficiency was at least 100-fold that of the nested PCR. The results suggest that L-PCR is advantageous for obtaining authentic DNA sequences from poorly preserved or recalcitrant ancient specimens.

## Introduction

Ancient DNA provides a profound perspective on the evolution and origin of organisms (Krause et al., 2006; Noonan et al., 2006; Larson et al., 2010; Thalmann et al., 2013; Zhang et al., 2013; Yang et al., 2020; Zhang et al., 2020). Discreetly acquiring authentic DNA sequences is a critical requirement for reliable phylogenetic deduction and evolutionary tracing. However, relentless processes such as oxidation, deamination, and depurination modify the nitrous bases and the sugar-phosphate backbone of the DNA (Hofreiter et al., 2001a; Hofreiter et al., 2001b; Uysal Yoca Ö. et al., 2021), invariably reducing the copy number of DNA recovered from ancient samples. These base lesions accumulate in DNA over time and may cause nucleotide misincorporations when ancient DNA sequences are replicated *in vitro* (Stiller et al., 2006; Gutaker and Burbano, 2017). Nuclear mitochondrial pseudogenes (NUMTs), which are generally analogous to homologous mitochondrial DNA (mtDNA) sequences, act as potential interference in ancient mtDNA analyses when they are inadvertently co-amplified with authentic mtDNA sequences (den Tex et al., 2010; Diroma et al., 2021). Since the first ancient DNA research was implemented (Pääbo, 1985), nested PCR has become a basic augmentation tool for retrieving low copy number DNA, along with uracil-N-glycosylase (UNG) pretreatment, which eliminates damaged templates (Willerslev and Cooper, 2005). However, in previous ancient DNA research, primer carryover from the first-stage PCR into the second-stage PCR could still have generated nonspecific amplification products, which would then require tedious authenticity verification. Therefore, the efficient and accurate retrieval of ancient DNA sequences faces a technical roadblock.

For high-fidelity amplification, complex primer design has received considerable attention. Therefore, a scheme for exploiting the loop-primer-meditated amplification mechanism was presented (Notomi et al., 2000). In particular, with the recognition of at least six conserved target DNA regions, loop-mediated isothermal amplification (LAMP) (Notomi et al., 2000; Tomita et al., 2008; Wong et al., 2018; Kashir and Yaqinuddin, 2020) and isothermal polymerase chain reaction (isoPCR) (Søe et al., 2013) have been extensively applied as diagnostic tools for nucleic acid tests (Huy et al., 2012; Murray et al., 2013; Park et al., 2018; Vichaibun and Kanchanaphum, 2020). This amplification mechanism can effectively and specifically boost outputs by forming stem-loop products with several target repeats.

**FIGURE 1:**
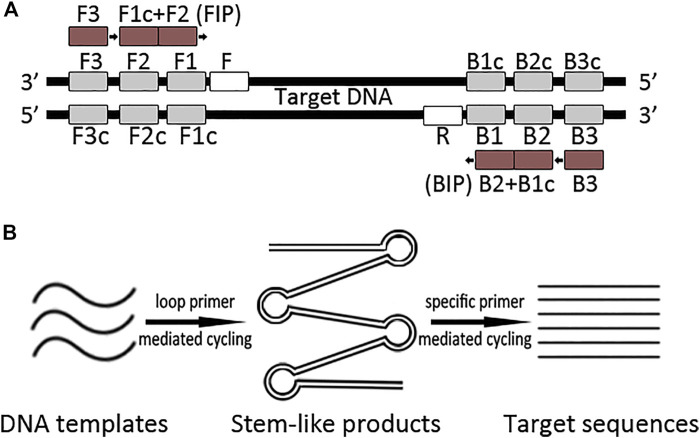
Schematic representation of the L-PCR assay. **(A)** Primers of the L-PCR assay contain a loop-primer set of four conserved primers which recognize six target DNA regions and a singleplex PCR primer set. **(B)** The L-PCR assay comprises two stages: the first stage uses a loop-primer set for specifically enriching a broader target region which comprises the target region; the second stage is a routine PCR process that uses a specific primer pair to obtain the target sequence.

Herewith, we supposed that incorporating complex primers into PCR reaction was helping to efficiently capture authentic poorly preserved DNA sequences. Therefore, we introduced loop primers to enrich ancient DNA templates in the PCR system, which we termed L-PCR. The theoretical procedure of L-PCR assay is illustrated in Figure 1. After optimizing the reaction system using modern pig and chicken samples for each primer set, the L-PCR assay was applied to amplify 66 ancient samples (including 27 pig samples and 39 chicken bones) and serially diluted modern pig DNA templates. The results were compared with those of nested PCR to evaluate the efficiency and accuracy of the newly created method.

## Materials and methods

### Modern sample and serially diluted DNA templates preparation

Five modern pig and five modern chicken blood samples were collected, respectively. The modern DNA were extracted following the standard protocol of the phenol-chloroform method. The DNA extractions were then quantified using Qubit 4 (Thermo Fisher Scientific) and applied to the subsequently optimization of amplification reaction for each primer set. The DNA samples of high quality and proper concentration were used to produce ten-fold dilution series, with concentrations ranging from 10^2^ ng/μl to 10^−6^ ng/μl. Serially diluted templates were used to evaluate the efficiency of the L-PCR assay.

### Ancient sample collection and DNA extraction

A total of 27 ancient pig bones or teeth and 39 ancient chicken bones were used in this study. To be specific, the pig samples were collected from three archeological sites from the middle Yellow River region including the ∼10,000-year-old Nanzhuangtou site, the ∼7,400-year-old Cishan site, and the ∼4,500-year-old Nanwa site, one ∼4,000-year-old site from the upper Yellow River region (Lajia), one ∼4,000-year-old site from the middle Yangtze River region (Qinglongquan). The chicken samples were collected from two archeological sites from the middle Yellow River region (Nanzhuangtou and Cishan), one site from the lower Yellow River region (the ∼4,000-year-old Wangyin site), and one site from the middle Yangtze River region (the ∼2,500-year-old Jiuliandun site). Detailed ancient sample information is shown in Table 1.

**TABLE 1:** Ancient sample information.

Species	Archaeological sites	Label	Sample number	Sample type	Location	Dates (YBP)
Pig	Nanzhuangtou	NZT	6	tooth/bone	Xushui County, Hebei Province	9,700–10,300
Pig	Cishan	CS	7	tooth/bone	Wuan County, Hebei Province	7,300–7,500
Pig	Nanwa	NW	3	Tooth	Dengfeng City, Henan province	4,000–5,000
Pig	Lajia	LJ	4	Tooth	Minhe County, Qinghai province	∼4,000
Pig	Qinglongquan	QLQ	7	tooth/bone	Yunxian County, Hubei province	3,900–4,400
Chicken	Nanzhuangtou	NZT	22	tibia/humerus/femur/tarsometatarsus	Xushui County, Hebei Province	9,700–10,300
Chicken	Cishan	CS	7	tibia/metatarsus	Wuan County, Hebei Province	7,300–7,500
Chicken	Wangyin	WY	6	tarsometatarsus	Yanzhou City, Shandong Province	3,500–4,500
Chicken	Jiuliandun Chu Tombs	JLD	4	left humerus	Zaoyang City, Hubei Province	2,300–3,000

Ancient DNA templates were prepared in a physically isolated laboratory. Ancient samples were prepared by cautiously cleaning the adhering soils, followed by washing with 5% sodium hypochlorite solution and double-distilled water and drying under ultraviolet irradiation. Ancient samples were then milled into powder, and 200–500 mg of powder was used for DNA extraction using the QIAamp^**®**^ DNA Investigator Kit (Qiagen). Amicon^®^ Ultra-4 (Millipore) was used to concentrate the ancient DNA solution to a volume of approximately 50 μl. The DNA extractions were quantified using Qubit 4 (Thermo Fisher Scientific).

### Primer design

The published mtDNA sequences of *S. scrofa* (AF486871) and *G. gallus* (NC001323) were used to design primers for obtaining the target pig or chicken mtDNA sequences, respectively. Primers for L-PCR were designed using the online Primer Explorer V4 software (http://primerexplorer.jp/e/index.html), which contained two outer primers (F3 and B3) and two inner primers (FIP and BIP). F3 and B3 were also used as the primers for the external round of nested PCR amplification. Then a pair of primers (F and R) between F3 and B3 was designed for the second round of nested PCR and L-PCR by Primer Primier 5. After amplification, F and R were also used as sequencing primers. In total, we generated two sets of primer pairs for pig mtDNA fragments of the cytochrome b gene (*Cytb*) and control region (CR), and three sets of chicken mtDNA fragments of the cytochrome oxidase I gene (*CO I*) and two fragments of the control region (CR). All primer sequences are shown in Table 2.

**TABLE 2:** Detail of PCR primers.

Species	Gene	Primer name	Sequence (5′-3′)	Primer length (bp)	Amplicon length (bp)	Annealing temperature(°C)
pig	Cytb	F3	CTT​CAT​AGG​CTA​CGT​CCT​GC	20	295	65
		B3	AGT​GTA​GTA​TGG​GTG​AAA​TGG​AAT	24		
		FIP (F1c + F2)	CCT​CAG​ATT​CAT​TCT​ACG​AGG​TCT​GTT​CTG​AGG​AGC​TAC​GGT​CAT​C	46		
		BIP (B1c + B2)	TGC​CAT​TCA​TCA​TTA​CCG​CCC​TTT​CCG​GTA​GGG​TTG​TTG​GA	41		
		F	CGG​AAC​AGA​CCT​CGT​AGA​ATG	21	142	61
		R	GGT​TTC​GTG​CAG​GAA​TAG​GA	20		
	CR	F3	CCT​GTG​TAC​GTC​GTG​CAT​TAA​C	22	251	65
		B3	TCA​TCA​ATA​GAA​ACC​CCC​ACG	21		
		FIP (F1c + F2)	GAT​ATG​TGC​TAT​GTA​CGA​TCC​CAT​GCA​TAT​AAG​CAT​GTA​C	41		
		BIP (B1c + B2)	CAC​GAG​CTT​AAT​TAC​CAT​GCG​AAG​AGG​GAT​CCC​TGC​CAA​G	40		
		F	TGC​TAG​TCC​CCA​TGC​ATA​TAA	21	179	61
		R	CCT​GCC​AAG​CGG​GTT​GCT​GG	20		
Chicken	CO I	F3	GCC​TCA​TCT​ACC​GTA​GAA​GC	20	260	65
		B3	GGT​AAG​GAG​AGG​AGT​AGT​AGG​A	22		
		FIP (F1c + F2)	TGG​CTA​GGT​CTA​CTG​ATG​CGC​GCA​CAG​GAT​GGA​CAG​TTT​AC	41		
		BIP (B1c + B2)	CAC​TAC​CAT​CAT​CAA​CAT​AAA​ACC​ATG​AGG​ACG​GAT​CAT​ACG​AAT	45		
		F	CCT​TTA​GCC​GGC​AAC​CTA​G	19	159	60
		R	TAG​GGG​TGT​TTG​GTA​TTG​TGA	21		
	CR1	F3	ACC​CAT​TAT​ATG​TAT​ACG​GGC​AT	23	333	65
		B3	CTG​AGA​CTG​GTC​ATG​AAG​TAC	21		
		FIP (F1c + F2)	CTG​TCT​ATG​GGG​AGG​GTA​AAT​GAG​CAC​ATT​TCT​CCC​AAT​GTC​C	43		
		BIP (B1c + B2)	CCA​TGT​TCT​AAC​CCA​TTT​GGT​TAT​GCT​GAT​CTC​TCG​TGA​GGT​G	43		
		F	CCA​TAG​ACA​GTT​CCA​AAC​CAC	21	186	62
		R	GGA​CGA​TCA​ATA​AAT​CCA​TCT​GAT	24		
	CR2	F3	CTA​TGA​ATG​GTT​ACA​GGA​CAT​AC	23	312	65
		B3	GAAGAGAGAAGATGCCGC	18		
		FIP (F1c + F2)	GAT​CTC​TCG​TGA​GGT​GGA​CGA​TTA​ACC​CAT​TTG​GTT​ATG​CTC​G	43		
		BIP (B1c + B2)	GCA​CAT​CCC​ATG​CAT​AAC​TCC​TGG​GAA​GAT​AAT​CCA​CAG​ATG​ACT	45		
		F	TGC​TCG​CCG​TAT​CAG​ATG​GAT	21	175	62
		R	AGT​TAT​GCA​TGG​GAT​GTG​CCT​G	22		

### Optimization of amplification reaction

Based on Notomi et al. (2000) and Tomita et al. (2008), the reaction system conditions including the Mg^2+^ concentrations (1.0 mM–3.0 mM), Taq DNA polymerase concentrations (0.5 U–2.0 U), concentration ratios between inner to outer primers (2:1 to 8:1), the annealing temperature for each primer set (55°C–70°C), and the input volume of first stage PCR products to the second stage amplifications (0.1 μl–1.0 μl), were detected to determine the optical reaction systems for both L-PCR and nested PCR. With the optical parameters, a singleplex PCR using real-time fluorescent SYBR Green reagents (Applied Biosystems) was conducted to quantitatively compare the efficiency of L-PCR to nested PCR on serially diluted DNA templates. Amplifications of the extraction blank controls and PCR blank controls were performed in all experiments to monitor contamination.

### Ancient DNA amplification

Ancient DNA extractions were amplified using both L-PCR assay and nested PCR. This resembled the optical reaction system except for special details, including 3 μl of ancient DNA extracts used in the PCR ingredients and 1U Uracil-N-glycosylase (UNG) was added to eliminate uracil from ancient DNA templates. All first-stage L-PCR and PCR reactions were initially incubated at 37°C for 10 min to facilitate the UNG, and were then performed under the cycling conditions with the optimal annealing temperatures. The subsequent singleplex PCR reactions were also executed under the aforementioned cycling conditions with their optimal annealing temperatures. All secondary amplified products detected by 2.5% agarose gel electrophoresis. The positive products were subjected to direct sequencing on an ABI 3730XL automated DNA sequencer using the BigDye Terminator v3.1 Kit (Applied Biosystems, Foster City, United States) and only samples with clear and intact sequencing histogram were regarded as successively achieved. Partial ancient samples were sent to the Ancient DNA Laboratory of the Research Center for Chinese Frontier Archaeology at Jilin University for independent replication.

### Clone and sequencing of ancient DNA PCR products

To test the authenticity of L-PCR amplification of ancient DNA, the amplification products of two positive ancient samples were analyzed by agarose gel electrophoresis (1% w/v) and were subsequently purified using Agarose gel Purification and Recovery Kit (Aidlab Co., Beijing, China). After that, the purified PCR fragments were ligated into the pBLUE-T vector (Aidlab Co., Beijing, China) to get multiplex clones, and eight clones for each sample were sequenced on an ABI 3730XL automated DNA sequencer (Applied Biosystems).

### Statistical analyses

The mean ± standard deviation was calculated for all data. The Wilcoxon test were used to compare the Ct value using SAS 9.2 (SAS Inst. Inc.). All values with *p* < 0.05 were regarded as being statistically significant.

## Results

### Establishment and optimization of amplification reaction system

By using modern pig and chicken DNA templates to determine the optical reaction system conditions for each primer set, the results showed that in a 25 μl reaction system 1.5 mM Mg^2+^ concentrations and 1U AmpliTaq Gold polymerase were suitable for all reactions. When the concentration of the inner primer was five times to the outer primer, it was expected to yield the most amplicons, and 0.2 μM forward and reverse outer primers was recommended in a 25 μl reaction system. After that, the optical annealing temperature for each primer set was determined (Table 2), which suggested that 64°C–66°C were suitable for all loop mediated primer sets and 65°C was chosen as the optical annealing temperature for the first stage L-PCR reaction of all primers. Moreover, serial volume products of the first stage amplification from 0.1 μl to 1.0 μl were input as the start template of the second stage amplification, and the result suggested 0.3 μl product input could yield most enriched targeting amplicons. To be specific, the optical reaction systems for L-PCR and nested PCR were descripted below.

For the L-PCR assay, the first-stage amplification using the loop primer set was carried out in a 25 μl reaction system containing 1U AmpliTaq Gold polymerase (Applied Biosystems), 1× PCR buffer concluded 3 mM Mg^2+^, 2 mM dNTPs, 1 μM forward and reverse inner primers (FIP and BIP), 0.2 μM forward and reverse outer primers (F3 and B3), and 0.5 μl DNA templates. The second-stage amplification was a singleplex PCR, in which 0.3 μl L-PCR product was used as the template, specific primers (F and R) were 0.5 μM each, and other ingredients were identical to the first stage. The L-PCR reaction was performed using the following cycling program: 94°C for 5 min, followed by 40 cycles of 94°C for 30 s, 65°C for 40 s, 72°C for 30 s, and a final extension of 10 min at 72°C. The subsequent singleplex PCR was performed under the same conditions except for the optical annealing temperature for each primer pair. For the nested PCR, the first-phase PCR was conducted with F3 and B3 primers using the optical annealing temperature for each primer set and the same ingredients as those used in L-PCR. For the second-phase PCR, 0.3 μl PCR product was added as a DNA template, and all other ingredients were identical to those in the first-phase PCR. The cycling programs were identical to those of singleplex PCR of L-PCR.

### Efficiency of L-PCR compared to nested PCR using serially diluted templates

A serially diluted DNA templates was used to test the enrichment efficiency of L-PCR assay and nested PCR with pig *Cytb* primer set. The results showed that good quality amplification products could be obtained by L-PCR for DNA templates with initial concentration of no less than 10° ug/μl, and even certain amplification products could be obtained with initial DNA templates concentration from 10^2^–10° pg/μl DNA templates by agarose gel analyses. Meanwhile, the results revealed that the nested PCR was able to achieve high quality amplification products with initial DNA templates concentration from 10^2^–10^2^ ug/μL DNA templates.

**FIGURE 2:**
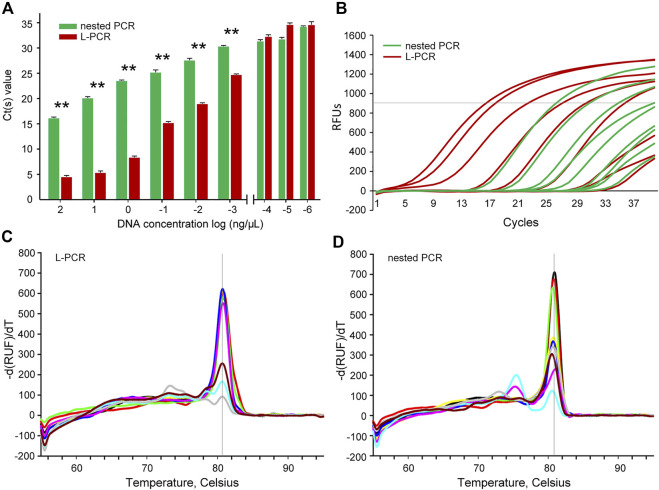
Analytical efficiency and accuracy between the nested PCR and L-PCR assay. **(A,B)** The Ct value comparison between the nested PCR and the L-PCR assay with serially diluted templates. **(C)** The melt temperatures for the L-PCR assay. **(D)** The melt temperatures for the nested PCR assay. The asterisk (*) indicates *p* < 0.05.

Moreover, the comparison of the quantitative determinations revealed that the L-PCR assay could reach the yield plateau at a lower Ct value (*p* < 0.01) than the nested PCR method at a template DNA concentration range of 10^2^ ng/μl to 10° ug/μl (Figure 2A). Within the limited 40 reaction cycles, L-PCR amplification with 10° ug/μl DNA templates reached a yield of 1,000 relative fluorescence units (RFUs), while at least 10° ng/μl DNA templates were needed for nested PCR to meet the same threshold (Figure 2B). Further, the L-PCR assay with an initial DNA concentration of 10° ng/μl could almost meet the equipotent yields of nested PCR with 10^2^ ng/μl DNA templates. These results demonstrated that the L-PCR assay could successfully detect total DNA in concentrations as low as 10° ug/μl and its productivity is at least 100-fold higher than that of conventional nested PCR.

### Determination of the accuracy of L-PCR assay

The melt curve analyses were carried out to detect the specificity of amplification at such low concentrations. The melt temperatures of the L-PCR second-stage amplification were clustered as one single sharp peak at approximately 80°C for the start DNA concentration of 10^2^ ng/μl to 10° ug/μl (Figure 2C), suggesting high fidelity of L-PCR assay. When the template concentration of L-PCR was in the case of undetectable dilutions, the melt temperature curves began to appear multi-peak and the peak value was low (Figure 2C), which suggested that the non-specific amplification increased, and reliability decreased. In contrast, the nested PCR had high amplification specificity only when the template concentration is 10° ng/μL or higher (Figure 2D). When the template concentration was lower than 10° ng/μl, the curve of melting temperature had two obvious peaks (Figure 2D), indicating that its specificity decreased with lower start template concentration than 10° ng/μl. This result supported the assumption that with multiple recognition regions on the target sequence, the L-PCR assay could perform well with rigorous and precise amplification beyond its limit of detection. For this, it was suggested that melt temperatures were not necessary to determine the fidelity while using the L-PCR assay, similar to the nested PCR (Uemura et al., 2008; Spielmann et al., 2019).

### Ancient sample amplification using nested PCR method and L-PCR assay

**FIGURE 3:**
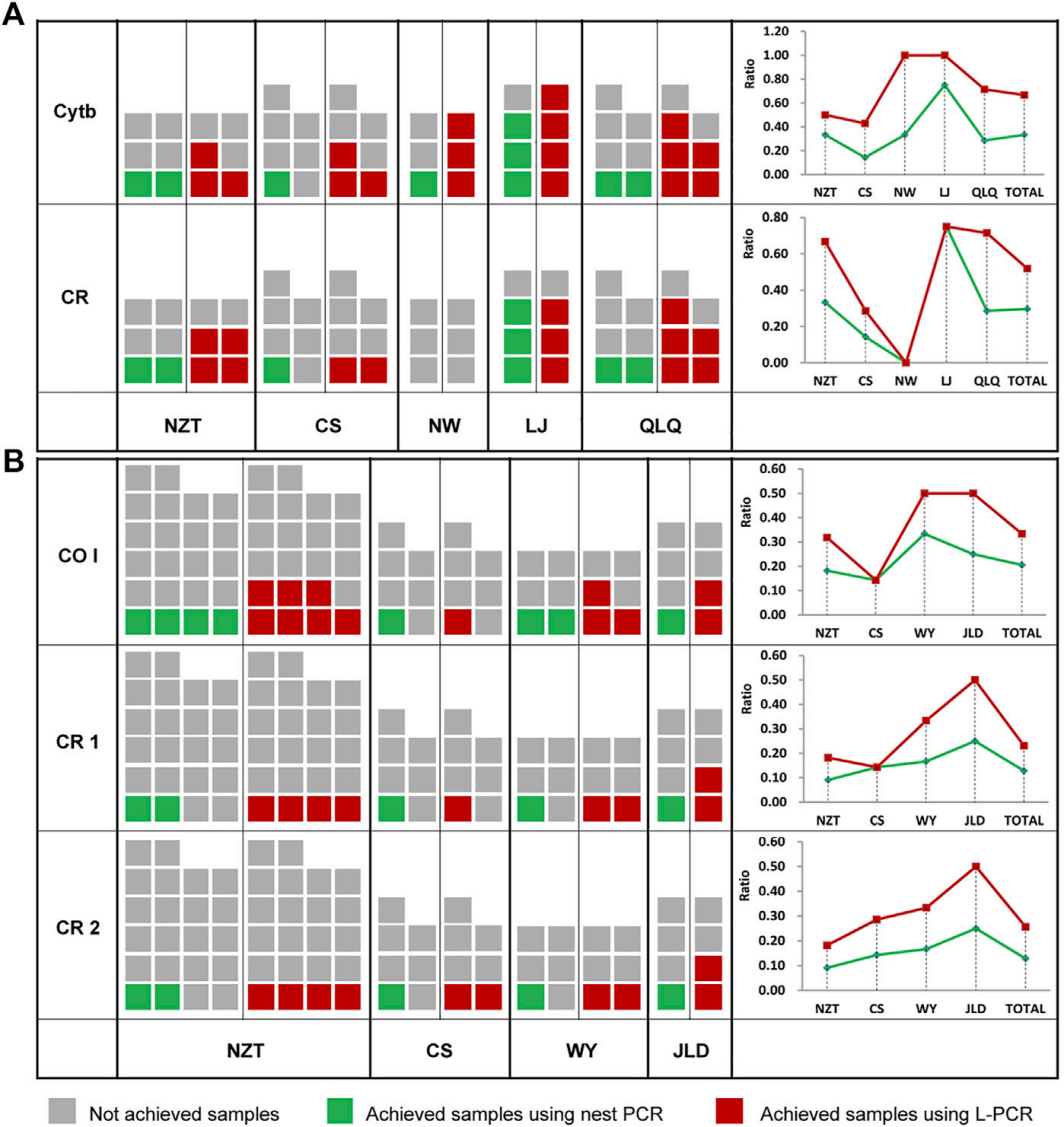
Comparison of ancient sample amplification using a nested PCR method and the L-PCR assay. **(A)** Numbers and ratios of ancient pig samples **(B)** Numbers and ratios of the ancient chicken samples.

In order to test the practical effect of L-PCR method in ancient DNA research, it was applied to amplify target sequences of *Cytb* gene and control region from ancient pig samples, and *COI* gene and control region fragments from ancient chicken samples. For both L-PCR and nested PCR, a weak correlation between the quality of ancient DNA templates and success of amplification. However, the results showed that the yield ratio did not decrease consistently with the increasing age in the overall comparison (Figure 3). Despite this, more sequences were achieved from specimens of ancient pigs than ancient chickens because chicken remains lack teeth and have a thinner bone wall. And both the numbers and ratios of the samples obtained using L-PCR assay were higher than the nested PCR, regardless of whether the DNA of pig and chicken samples came from as DNA templates (Figure 3).

### Clone and sequencing of ancient DNA PCR products

**FIGURE 4:**
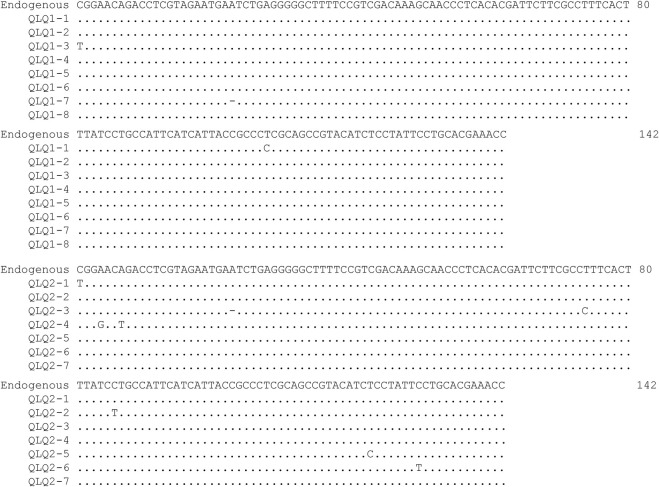
Alignment of the achieved *Cytb* clones of two Qinglongquan pig samples using L-PCR without UNG treatment. The “Endogenous” sequence is determined by the results of amplification after UNG treatment. Nucleotides identical to the endogenous sequence are indicated by dots.

Two ancient pig samples from the ∼4,000-year-old Qinglongquan Site were amplified by L-PCR with *Cytb* gene primer. A total of eight clones for each sample were sequenced and the multiple alignment showed that only a few damage sites were found in different clones of the two samples and the consensus sequence was identical to the endogenous sequence (Figure 4), indicating that L-PCR had high fidelity to ancient DNA research. However, since some damage sites did exist in different clones, it should be recommend adding proper UNG enzyme in the first amplification reaction system of L-PCR to eliminate possible damage templates.

## Discussion

Previous storage experiments have revealed that diluted DNA templates are more prone to drop under the minimum fragment threshold than concentrated DNA, even after a short period of storage. Therefore, the serially diluted DNA templates were used to simulate the degradable and fragmented ancient DNA templates; the results demonstrated that the L-PCR assay was efficient and accurate for ancient DNA amplification (Figure 2).

Currently, the use of UNG and multiple clones after amplification are conventional troubleshooting methods for assuring the authenticity of ancient DNA sequences. However, eliminating damaged templates using UNG also greatly aggravates the dilemma of the paucity of ancient DNA and the proposed after-amplification authenticity validation, such as multiple clones, requires increased time inputs but with fewer data outputs. Primers used in L-PCR were recognized with six distinct conserved regions on the target sequence, giving rise to amplification of the target sequence with high selectivity from the beginning, excluding damaged templates, and eliminating false amplification of possible homologous NUMTs or other contaminants. Similar to LAMP, the first-stage amplification of the L-PCR assay generated stem-loop DNAs with several inverted repeats and cauliflower-like structures (Notomi et al., 2000; Hardinge and Murray, 2019), thereby providing a very high molecular DNA nanostructure without primer carryover and avoiding false positives due to base lesions (Briggs et al., 2010; Hardinge and Murray, 2019).

Hostile sample deposit conditions, including high or fluctuating temperatures, excessive acidity, inappropriate salinity, and immoderate water percolation, easily leads to DNA lesions, such as strand breaks, oxidative lesions, cross-links, and other unknown types of damage in ancient DNA. Fragmentation and DNA modifications therefore hindered the elongation of DNA strands by DNA polymerase (Pääbo et al., 2004; Berdis, 2017; Garafutdinov et al., 2020). Thus, the DNA preservation conditions are of great relevance in obtaining target DNA in ancient DNA research. In the present study, the deposit conditions of all archeological sites were pre-investigated; therefore, we designed amplicons with variable lengths and different regions. However, our results suggested that the yield ratio did not decrease consistently with increasing age. Moreover, regardless of whether DNA templates came from younger or older pig and chicken samples, the L-PCR assay were able to yield higher numbers and ratios of ancient DNA sequence from the samples. With L-PCR enrichment of ancient templates, the L-PCR assay presents an effective method of generating valuable sequences in ancient DNA research.

In addition, single nucleotide polymorphism (SNP) sites have been applied as DNA markers in, for example, species identification in forensic detection (Hou et al., 2014; Jiang et al., 2020; Sinha et al., 2020), traditional Chinese medicine research (Cao et al., 2014; Hou et al., 2017), and agricultural product quality control (Fukuta et al., 2004; McClure et al., 2018; Raatz et al., 2019). However, objects investigated in these fields are usually characterized by unknown composition, seriously damaged DNA, or DNA that is degraded to low copy numbers over time or under multifarious processes. These intrinsic challenges generally limit the utilization of conventional detection approaches. With high productivity and high fidelity, the L-PCR assay can be regarded as a tool that addresses these challenges.

In conclusion, the L-PCR assay combines the merits of PCR and loop primer-mediated amplification. Possessing high efficiency and fidelity, this assay is an effective tool for obtaining fragmented but valuable authentic DNA sequences from poorly preserved or recalcitrant ancient specimens. It can also be applied to improve molecular diagnosis using poor DNA templates.

## Data Availability

The datasets presented in this study can be found in online repositories. The names of the repository/repositories and accession number(s) can be found below: DRYAD (http://datadryad.org) with DOI https://doi.org/10.5061/dryad.zw3r228b4.
